# Salt tolerance is evolutionarily labile in a diverse set of angiosperm families

**DOI:** 10.1186/s12862-015-0379-0

**Published:** 2015-05-19

**Authors:** Camile Moray, Xia Hua, Lindell Bromham

**Affiliations:** Division of Ecology, Macroevolution and Macroecology, Evolution and Genetics, Research School of Biology, Australian National University, Brinkin, 0200 Australia

**Keywords:** Angiosperms, Comparative method, Halophyte, Macroevolution, Repeated evolution

## Abstract

**Background:**

Salt tolerance in plants is rare, yet it is found across a diverse set of taxonomic groups. This suggests that, although salt tolerance often involves a set of complex traits, it has evolved many times independently in different angiosperm lineages. However, the pattern of evolution of salt tolerance can vary dramatically between families. A recent phylogenetic study of the Chenopodiaceae (goosefoot family) concluded that salt tolerance has a conserved evolutionary pattern, being gained early in the evolution of the lineage then retained by most species in the family. Conversely, a phylogenetic study of the Poaceae (grass family) suggested over 70 independent gains of salt tolerance, most giving rise to only one or a few salt tolerant species. Here, we use a phylogenetic approach to explore the macroevolutionary patterns of salt tolerance in a sample of angiosperm families, in order to ask whether either of these two patterns – deep and conserved or shallow and labile - represents a common mode of salt tolerance evolution. We analyze the distribution of halophyte species across the angiosperms and identify families with more or less halophytes than expected under a random model. Then, we explore the phylogenetic distribution of halophytes in 22 families using phylogenetic comparative methods.

**Results:**

We find that salt tolerance species have been reported from over one-third of angiosperm families, but that salt tolerant species are not distributed evenly across angiosperm families. We find that salt tolerance has been gained hundreds of times over the history of the angiosperms. In a few families, we find deep and conserved gains of salt tolerance, but in the majority of families analyzed, we find that the pattern of salt tolerant species is best explained by multiple independent gains that occur near the tips of the phylogeny and often give rise to only one or a few halophytes.

**Conclusions:**

Our results suggest that the pattern of many independent gains of salt tolerance near the tips of the phylogeny is found in many angiosperm families. This suggests that the pattern reported in the grasses of high evolutionary lability may be a common feature of salt tolerance evolution in angiosperms.

**Electronic supplementary material:**

The online version of this article (doi:10.1186/s12862-015-0379-0) contains supplementary material, which is available to authorized users.

## Background

Only 1 - 2 % of angiosperm species are known to be halophytes, able to live and reproduce in saline soils [1, 2]. The rarity of salt tolerance is unsurprising considering it is a costly and complex ecological strategy; halophytes may have modifications to many parts of their physiology and anatomy in order to combat the damaging effects of osmotic and metabolic stress, which can cause impaired growth and reproduction [2–4]. However, halophytes are found in a wide range of angiosperm families and they occupy diverse habitats worldwide.

Salt tolerance has also clearly evolved multiple times in angiosperms [5, 6]. The evolutionary patterns of salt tolerance in plants have been studied in detail in only a few taxonomic groups, and these studies have revealed two very different patterns of salt tolerance evolution. In one well-studied group, the chenopods (Chenopodiaceae), salt tolerance appears to be phylogenetically conserved [7], arising only once or twice in the history of the group, then being retained in a large proportion of species in the family. In contrast, a study on the grass family (Poaceae) estimated that there have been at least 70 origins of salt tolerance within the family [8]. Most of these inferred origins were near the terminal taxa (tips) of the phylogeny, suggesting multiple shallow origins, each giving rise to only one or a few salt tolerant species. The pattern of salt tolerance evolution inferred in the Poaceae is interesting because it suggests that, at least in the grasses, salt tolerance has evolved repeatedly in a range of lineages, despite the complexity of salt tolerance adaptations. However, the observation that salt tolerance does not persist over long evolutionary timescales in the grasses may indicate that while salt tolerance is easy to gain, it is also frequently lost through trait reversal or extinction, implying that there are costs associated with the adoption of salt tolerance.

These two different phylogenetic patterns suggest very different macroevolutionary dynamics. Salt tolerance is highly conserved in the chenopods, with a large number of salt tolerant species arising from only a few independent origins. But in the grasses, salt tolerance is highly labile, in the sense that it is gained and lost relatively frequently. Which, if either, of these patterns is observed in other families of angiosperms? To answer this question, we use a phylogenetic comparative approach to investigate and characterize patterns of halophyte diversity and evolution among angiosperm families.

Halophytes use a variety of physiological and anatomical traits to survive in saline habitats, and these traits can vary between species. Some halophytes exhibit complex anatomical modifications like salt glands or hairs, but most halophytes rely on osmotic regulation, modifying existing physiological mechanisms to mitigate salinity levels within the plant [9, 10]. These strategies can also vary amongst closely related halophytes and among halophytes that occupy similar habitats, for example the differential presence of succulence among closely related chenopods [7] or salt glands among phylogenetically diverse mangrove species [11]. Instead of identifying specific environmental or physiological differences between halophytes, we focus on the broad distribution of salt tolerance as an ecological strategy amongst angiosperms, at the family and species levels. We first examine how halophytes are distributed among the angiosperm families, identifying any families that have more or less halophytes than expected by chance. Then we use a number of phylogenetic measures to analyze the observed evolutionary patterns of salt tolerance in a sample of 22 angiosperm families. This sample includes large families with many known halophytes, including families with both more and less halophytes than expected.

## Results

### Halophyte Diversity

We found that the observed distribution of halophytes across angiosperm families was significantly nonrandom (*p* < 0.001). Of the 411 families included in the taxonomic analysis, 146 families have one or more known halophytes (See Methods and Additional file 1). We found that 51 of the 411 families have significantly more halophytes than expected by chance; examples include Amaranthaceae, Poaceae and Rhizophoraceae (see Additional file 1: Table S1). 68 families have significantly fewer halophytes than expected by chance, for example Acanthaceae, Lamiaceae and Fabaceae.

### Evolutionary Patterns

**Fig. 1:**
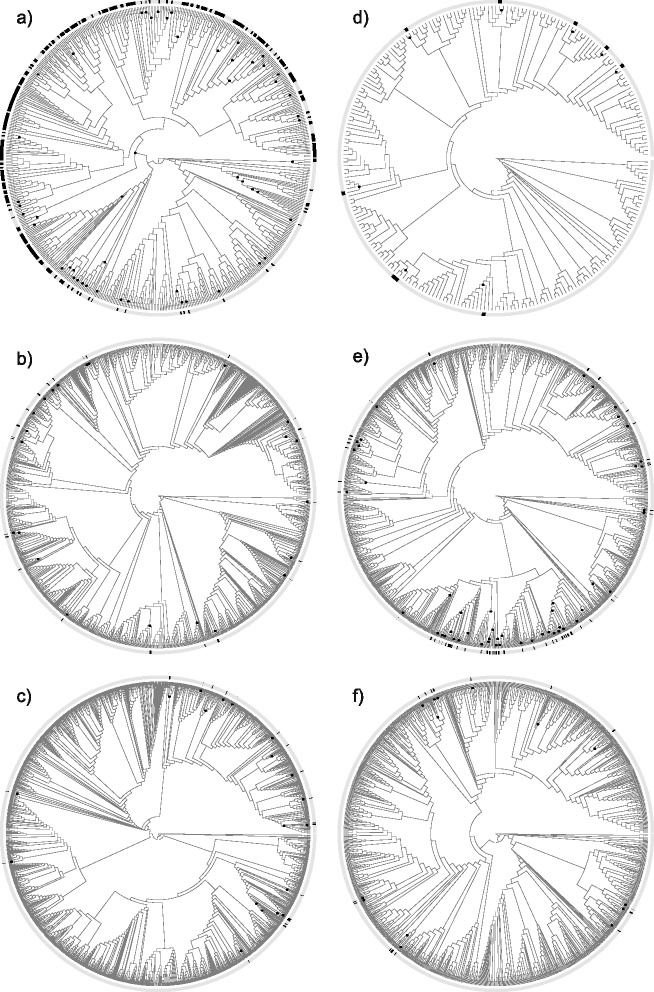
Family subtrees for a sample of six of the families analyzed with significant NoTO and/or SSCD values. Inferred gains of salt tolerance (see Methods) are marked on each family with black circles. Tips in the subtrees identified as halophytes are marked in black in the ring around the subtree. The subtrees represent **a**) Amaranthaceae, **b**) Apiaceae, **c**) Brassicaceae, **d**) Cucurbitaceae, **e**) Cyperaceae, and **f**) Euphorbiaceae. All 22 subtrees included in the analysis are presented in Additional file 1: Figure S1. Subtree plots were created using the “Diversitree” package [58]

For each family analyzed, we created a family subtree that included all tips from a large angiosperm phylogeny [12] belonging to each family according to GenBank taxonomy (See Methods). In the 22 family subtrees analyzed, we observed a range of evolutionary patterns of salt tolerance (see Fig. 1 and Additional file 1: Figure S1). In general, evolutionary gains of salt tolerance appeared close to the tips, across the family subtrees. One measure used to assess the evolutionary patterns of salt tolerance across families was the number of tips per origin (NoTO), the average number of taxa descending from each inferred evolutionary origin of salt tolerance in a family. The median value of NoTO across all 22 families analyzed was 1.3 and nineteen of the family subtrees had a NoTO value less than two. This observation indicates that the inferred gains of salt tolerance in these family subtrees typically give rise to less than two descendant halophyte tips. In contrast, a few families (Rhizophoraceae, Amaranthaceae and Tamaricaceae) had higher NoTO values than the other families, meaning that each gain of salt tolerance in these families is deeper in the subtrees and leads to comparatively larger clades of halophytes than observed in the other families analyzed. Tamaricaceae was the only family in our sample with significantly fewer salt tolerance gains than expected given the number of known halophytes and halophytic taxa were significantly clustered.

Over half of the families analyzed had a similar phylogenetic distribution to the pattern found in the grasses [8]. In these families, given the observed number of halophytes in each of the family subtrees, either 1) salt tolerance has evolved more times than expected under a Brownian motion model (significantly lower NoTO) and/or 2) clades of halophytes are less clustered than expected under Brownian motion model of trait evolution (significantly higher sum of sister clade differences (SSCD), see Methods) (Table 1). When comparing with the results from the angiosperm diversity analysis, we found that this labile evolutionary pattern is found in families with varying proportions of halophytes, including those with more, fewer, and within the expected number of halophytes based on family size.

**Table 1:** Results of taxonomic and phylogenetic analyses for a sample of 22 angiosperm families

Order	Family	Family size	Known Halophytes	Halophytes in family (%)	Taxonomic pattern	Family subtree size	Species in subtree (%)	Halophytes in subtree	Halophytes in subtree (%)	Halophytes sampled in subtree (%)	Inferred origins	NoTO	NoTO (*p*)	SSCD (*p*)
Apiales	Apiaceae	3780	33	0.9		1082	28.6	26	2.4	78.8	22	1.2	**0.00**	**0.00**
Arecales	Arecaceae	2361	35	1.5	more	415	17.6	19	4.6	54.3	15	1.3	0.06	**0.00**
Asterales	Asteraceae	23600	267	1.1		4618	19.6	97	2.1	36.3	87	1.1	**0.00**	**0.00**
-	Goodeniaceae	430	6	1.4		69	16.0	6	8.7	100	6	1.0	0.09	**0.01**
Brassicales	Brassicaceae	3710	38	1.0		1355	36.5	21	1.5	55.3	19	1.1	**0.00**	**0.00**
Caryophyllales	Amaranthaceae	2275	507	22.3	more	613	26.9	262	42.7	51.7	54	4.9	0.16	**0.00**
-	Tamaricaceae	90	55	61.1	more	42	46.7	29	69.0	52.7	1	29.0	*1.00*	*1.00*
Cucurbitales	Cucurbitaceae	960	14	1.5		247	25.7	9	3.6	64.3	8	1.1	0.14	**0.02**
Ericales	Primulaceae	2590	14	0.5	fewer	546	21.1	8	1.5	57.1	5	1.6	0.65	0.55
Fagales	Casuarinaceae	95	12	12.6	more	88	92.6	12	13.6	100	7	1.7	0.46	0.08
Gentianales	Rubiaceae	13150	13	0.1	fewer	1393	10.6	7	0.5	53.8	7	1.0	0.09	**0.01**
Lamiales	Acanthaceae	4000	18	0.5	fewer	498	12.5	9	1.8	50.0	5	1.8	0.75	0.54
-	Lamiaceae	7173	27	0.4	fewer	941	13.1	14	1.5	51.9	11	1.3	0.14	0.05
Malpighiales	Euphorbiaceae	5735	42	0.7	fewer	1047	18.3	16	1.5	38.1	14	1.1	**0.03**	**0.00**
-	Rhizophoraceae	149	19	12.8	more	40	26.8	18	45.0	94.7	6	3.0	0.52	0.71
Myrtales	Combretaceae	500	12	2.4	more	25	5.0	8	32.0	66.7	6	1.3	0.23	0.25
-	Lythraceae	620	21	3.4	more	119	19.2	14	11.8	66.7	8	1.8	0.53	0.46
-	Myrtaceae	4620	47	1.0		612	13.2	20	3.3	42.6	19	1.1	**0.00**	**0.00**
Poales	Cyperaceae	5430	121	2.2	more	1087	20.0	57	5.2	47.1	52	1.1	**0.00**	**0.00**
-	Juncaceae	430	22	5.1	more	124	28.8	12	9.7	54.5	8	1.5	0.31	0.45
-	Poaceae	11160	335	3.0	more	2291	20.5	173	7.6	51.6	127	1.4	**0.00**	**0.00**
Rosales	Rosaceae	2520	9	0.4	fewer	1010	40.1	8	0.8	88.9	8	1.0	0.05	0.05

Family and order names are based on APG III [48]. Family size is the mean estimated number of species in the family reported on the APG III website [49]. The halophytes column lists the number of known halophytes species in each family. Family subtree size represents the number of taxa in the phylogenetic tree used for analysis, and halophytes in subtree is the number of known halophytes included in each family subtree. The halophytes sampled in subtree represents the percent of known halophytes that are present in each family subtree. The taxonomic pattern column identifies families with more or fewer halophytes than expected by chance based on the taxonomic analysis (see Methods). The results of the metrics used to distinguish evolutionary patterns of salt tolerance are presented. For the number of tips per origin (NoTO), *p*-values represent whether the average number of halophytes arising from each inferred gain of salt tolerance is smaller expected under Brownian motion (*p* < 0.05). For the sum of sister clade differences (SSCD), *p*-values represent whether halophytes are less clustered than expected under Brownian motion (*p* < 0.05). Test statistics that are significantly different to the null model are presented in bold. Significant results for Tamaricaceae are italicized to highlight that this is the only significantly conserved pattern of salt tolerance, where significantly more tips per gain and a significantly smaller SSCD

Correlation tests suggest that there is no significant association between taxon sampling proportion and estimates of SSCD (*p* = 0.660, τ = 0.071) or NoTO (*p* = 0.728, τ = 0.056) estimates. The proportion of halophytic taxa in the subtrees is not correlated with SSCD (*p* = 0.158, τ = 0.230) or NoTO *p*-values (*p* = 0.087, τ = 0.270).

## Discussion

Using a list of known halophytes assembled from a range of published sources, we find that one-third of angiosperm families contain species reported as being able to live in saline conditions. We show that the distribution of salt tolerant species among angiosperm families is not consistent with a random distribution, and that some families have significantly more halophytes than expected given the family size, while others have significantly fewer salt tolerant species.

Not only does the proportion of halophytes differ between families, but the phylogenetic distribution of salt tolerance within families also varies. Specifically, we set out to test whether the pattern of salt tolerance in grasses (Poaceae) – with many, shallow, scattered gains – was also found in other families. A few families show the opposite pattern, where salt tolerance has been gained deep in the family and retained by a large proportion of descendants [7]; examples include Tamaricaceae which contains the highly salt tolerant salt cedars and many species with specialized anatomical traits like salt glands [13]; Amaranthaceae, which includes the halophyte-rich groups formerly classified under Chenopodiaceae; and Rhizophoraceae, which contains many mangrove species. However, over half of the families analyzed show a pattern like the grasses, consistent with many, shallow gains of salt tolerance. The fact that we find this labile pattern of salt tolerance evolution in a phylogenetically diverse set of families with different proportions of halophytes suggests that the observed pattern of many independent gains of salt tolerance is not simply explained by the proportion of halophytes in a group.

One limitation of broad comparative analyses like this one is that we can only gain information from data on known halophytes and sequenced angiosperm taxa. Specifically, the limitations in data used in this study come from two main sources. One problem is the incidence of false negatives in the halophyte list. Most published lists of halophytes are based on observational data, and there are likely to be other salt tolerant species that have not been described in the literature or included in published lists. For example, there are likely many species living in non-saline habitats that have the capacity for salt tolerance but have not yet been formally tested. One solution to improve future analyses is to move away from list-based methods drawn from single species experiments and observational data. For example, it may be possible for phylogenetic studies to contribute to the identification of salt tolerance lineages, for example by using use phyloprediction [14] or geochemical modeling to identify lineages that are likely to be salt tolerant [15, 16].

A second potential source of error is incomplete phylogenetic sampling. The phylogeny used in this study includes about 20 % of known angiosperm species, so there are some known salt tolerant taxa that are not included in this tree (see Table 1 for details). Correlation tests did not indicate any consistent effect of the proportion of total species sampled in a family or of the proportion of halophytes in the subtrees on the results of NoTO and SSCD, suggesting that sampling proportion does not significantly influence the results of our analysis. Increased sampling is unlikely to change the overarching pattern because salt tolerant taxa in most family subtrees with significant NoTO and SSCD are sparsely distributed and many of the inferred gains are distantly related on the trees (see Fig. 1 and Additional file 1: Figure S1). This pattern suggest that in most cases adding more salt tolerant taxa is likely to either increase the total number of inferred salt tolerance gains (if adding species that are not closely related to known halophytes) or maintain the number of inferred gains (if adding to a clade of known halophytes). However, there are some clades, for example in Cyperaceae and Amaranthaceae, with denser groups of halophytes, where the number of inferred gains relative to the number of halophytes is more likely to be reduced by adding more halophytes (Fig. 1a,f). Similarly, removing identified halophytes with only low or seasonal tolerance to salinity could in some cases increase phylogenetic clustering of halophytes, reducing the number of inferred gains (if removing species that are not closely related to known halophytes), or break up some clades, possibly increasing the estimated number of gains. Based on the extant pattern of halophytes, our analysis suggests that salt tolerance has been gained at least 600 times in the 22 families analyzed. And if we assume that each of the other angiosperm families that contain halophytes also represents at least one independent gain of salt tolerance, there are likely to be 124 additional gains or more across the angiosperms.

Our results are consistent with the findings of two previous group-specific studies on phylogenetic patterns of salt tolerance [7, 8]. We infer over 100 gains of salt tolerance within the grass family subtree, and confirm that halophytes are more phylogenetically dispersed than expected under Brownian motion. We also demonstrate that the Amaranthaceae has relatively high numbers of species per inferred gain, indicating a more conserved pattern of salt tolerance evolution compared to the other families in the analysis. While we estimate that salt tolerance is significantly less clustered than Brownian motion in Amaranthaceae, this result appears to be driven by about one-third of the family with notably fewer halophytes than the rest of the tree (Fig. 1a).

Given that salt tolerance may involve many anatomical, physiological and life history modifications, it may seem surprising that it has evolved so many times in such a wide range of lineages. However, it has been suggested that other stress tolerance strategies involving complex sets of ecophysiological traits have also evolved multiple times [17–20]. One explanation for how salt tolerance has evolved multiple times is that the required physiological or anatomical changes can build on precursor traits acquired earlier in the history of the lineages. A well-studied example of how complex physiological traits can build on precursor traits is C_4_ photosynthesis in the grasses [21]. In a few angiosperm families researchers have inferred many independent evolutionary origins of C_4_ photosynthesis, a specialized form of photosynthesis often associated with arid-adapted lineages [19, 22, 23], which requires many biochemical and anatomical modifications. Lineages with a higher proportion of vascular bundle sheath cells have a higher frequency of evolution of C_4_ photosynthesis, suggesting that some types of foliar anatomy facilitate the transition to C_4_ [23]. Similarly, if salt tolerance builds on existing physiological or anatomical traits, then a lineage with these traits may have a higher likelihood of giving rise to halophytic species. For example, C_4_ grass lineages are more likely to contain halophytes than C_3_ lineages, possibly because C_4_ photosynthesis allows more efficient water use and therefore limits the impact of salinity by reducing the uptake of ions and limiting the effects of osmotic stress [24].

Although the idea of evolutionary precursors may explain why some lineages develop salinity tolerance more often than others, the question remains: why are salt tolerant lineages often found as singletons on the phylogeny or in small clades? There are several broad explanations for this pattern, which are not mutually exclusive. One explanation is that the observed distribution of halophytes could reflect patterns of change in land salinity over time. Although some saline areas are long lived (*e.g.*, coastal habitats), in some areas salinity can vary over small spatial scales or shift on a seasonal basis [25]. If lineages are rapidly responding to changing salinity, this could partly explain why we infer mostly shallow gains of salt tolerance that give rise to only one or a few extant halophytes. However, recent origins of saline habitats is unlikely to provide a general explanation for the multiple recent gains of salt tolerance in many families, because some saline habitats are stable over long evolutionary time periods, so should provide persistent habitat for saline specialists.

Another explanation for why there are so many small clades of halophytes is that salt tolerance may be a costly ecological strategy that is relatively easy to gain but difficult to maintain. For example, high plasticity could enable some lineages to transition into harsh or novel habitats over evolutionarily short time scales [26, 27]. However, maintaining a strategy like salt tolerance could be physiologically costly, for example due to the cost of producing osmoprotectants or increasing investment in reactive oxygen species scavenging and antioxidant production (reviewed in [28]). The high physiological cost of salt tolerance could lead to increased extinction rates in halophytes, or high reversal rates if lineages that invest less in salt tolerance mechanisms have a competitive advantage. This scenario could lead to an extant pattern of many shallow gains of salt tolerance dispersed across the phylogeny. Some research suggests that the more salt tolerant a species, the less competitive it is in less saline or non-saline environment [29, 30], although the generality of these claims are disputed [31]. Reduced competitive ability may threaten the persistence of halophytes if land salinity subsides, and halophytes may not be ecologically competitive when transitioning back into a non-saline environment [32], which could lead to local extinction or the loss of salt tolerance. However, the lower competitive ability may not always be a direct result of salt tolerance [31], and high salinity tolerance may even confer a competitive advantage for some species in non-saline habitats. For example, salt cedars (*Tamarix*, Tamaricaceae) are highly salt tolerant, yet they are invasive in some non-saline and low-saline riparian habitats. Salt cedar populations are capable of displacing natives by using more water and excreting salt into the soil, creating a toxic environment for non-salt tolerant native species [33].

It is unlikely that either changes in land salinity patterns or the cost of salt tolerance can fully explain why salt tolerance has evolved many times and why halophytes are often found as singletons and in small clades. And it has been suggested that, in general, the transition into different habitats and the evolution of ecological traits may be highly context dependent [21]. Identifying the phylogenetic patterns of salt tolerance represents an important step towards understanding salt tolerance evolution. We hope that reporting results in the context of angiosperm families will be useful for more detailed studies in future on the environmental and physiological aspects of salt tolerance evolution in different lineages. In future it would be interesting to explore the role that related traits, order of trait acquisition, and climatic history have played in the observed patterns of salt tolerance evolution, as has been examined for C_4_ photosynthesis [23] and freezing tolerance [34].

## Conclusions

Salt tolerance in plants is an interesting case study in macroevolution [35]. Salt tolerance is an ecological strategy that often involves complex physiological features. Halophytes are rare, yet they are found in a diverse set of taxonomic groups. Our analysis shows that in a range of angiosperm families, salt tolerance has been gained a surprising number of times and that these transitions are shallow and spread out near the tips of the phylogeny. This suggests that while the evolutionary pattern of salt tolerance varies across angiosperm families, it seems that salt tolerance can evolve frequently in many different genetic backgrounds. This result is intriguing, given how difficult it has to been to manipulate the salt tolerance of commercial crop varieties [31, 36], but the frequent evolution of salt tolerance may give hope that many plant lineages can build on existing physiological and anatomical traits to develop increased tolerance of environmental salinity.

## Methods

### Halophyte database

Our aim was to broadly investigate the patterns of salt tolerance distribution as an ecological strategy across angiosperms. We first compiled a list of known halophytes. Instead of differentiating halophytes based on specific traits or environmental conditions, we analyzed salt tolerance as a binary trait, categorizing plants as reported to tolerate salt (labeled as 1) or not reported as salt tolerant (labeled as 0). Analyzing salt tolerance as a binary character is the only practical approach for a broad scale comparative study since there are relatively few species for which we have information on specific levels of salt tolerance, and this approach also allowed us to study a wide variety of salt tolerant species. We started with a published list of approximately 2600 taxa observed in saline habitats [37]. We then searched the literature and added taxa from five additional halophyte lists that were published more recently [38–42] (See Additional file 1 for details). These published lists included halophytes identified from field surveys and observational data. It is possible that some taxa included in these lists have low salinity tolerance, are only tolerant to limited exposure to salinity (*e.g.*, seasonal salinity), or have experienced acclimation to salinity [43, 44]. For this study, we consider that these species have an underlying propensity for developing salt tolerance, and so their inclusion is useful in a broad study on the evolution of salt tolerance. The resulting list contained 4515 taxa reported to be salt tolerant (including infraspecific taxa). We then searched for synonyms and accepted names of each taxon in this list according to The Plant List [45] using the R package ‘taxonstand’ [46]. Because the taxonomic and phylogenetic analyses had different aims, we created separate lists for each analysis, which are described below.

### Halophyte Diversity

Our first aim was to investigate the taxonomic distribution of halophytes across angiosperm families. Although families may represent lineages of different ages or evolutionary patterns, here they are used simply as a convenient taxonomic division of angiosperm diversity into defined groups. We identified 411 unique angiosperm families by checking the 413 families recognized by the Linear Angiosperm Phylogeny Group (LAPG III) [47] against the APG III website [48, 49] (two were found to have equivocal names, see Additional file 1). We then collected mean estimates of species numbers for each of the 411 families from the APG III website [49], totaling 276,000 species. Since these family size estimates are reported at the species level, we needed to compile a list of halophytic species. We selected only the unique set of accepted halophyte species names according to The Plant List [45]. We collapsed the names of accepted infraspecific taxa to the species level, counting a species as salt tolerant if one of its varieties or subspecies was listed as a halophyte. The resulting list contained 2852 unique halophyte species (see archived data for halophyte list). We then counted the number of known halophyte species in each angiosperm family.

Then, we tested if halophytes were distributed randomly across families. We tested whether the number of halophytes in each family followed a binomial distribution parameterized by family size and the probability of being salt tolerant equal to the observed proportion of halophytes over all the angiosperm families. We applied a G-test of independence to estimate the overall fit of the model to the angiosperm families, using the likelihood.test function in R package “Deducer” [50].

We then calculated the probability of observing the number of known halophytes for each family based on the binomial distribution. This probability allowed us to identify families with significantly more or fewer halophytes than expected by chance. If the probability of a family having the same number or more halophytes than observed is lower than 0.05, the family is considered to have significantly more halophytes than expected by chance. If the probability of a family having the same number or fewer halophytes than observed is lower than 0.05, the family is considered to have significantly fewer halophytes than expected by chance.

### Evolutionary patterns

#### Phylogenies

Our second aim was to investigate the phylogenetic patterns of halophyte distribution. Because halophytes are rare, we did not analyze the distribution of salt tolerance across the entire angiosperm phylogeny, but focused on a subset of families that each contained many halophytes. Our main aims in family selection were to: (1) collect the largest families containing a sufficient number of halophytes to provide sufficient power for the analysis of evolutionary patterns; and (2) select families that were found to have more, fewer or within the expected range of halophytes in the taxonomic analysis, so as not to bias the analysis to families with a higher representation of salt tolerant taxa. All our analyses were conducted using phylogenetic information from the largest available phylogeny for angiosperms [12]. This phylogeny includes all appropriate angiosperm sequences on GenBank, including infraspecific taxa, which covers approximately 20 % of angiosperm species.

We first assigned each tip in the phylogeny to a family according to GenBank taxonomy using the TaxoGB function in the R package “BoSSA” [51], and recorded the total number of tips (terminal taxa in the phylogeny) associated with each family. We then determined which tips in the phylogeny were halophytes, based on whether the tip name was included in either the halophyte names presented in the original publications or the accepted names found in the synonymy search (total 5030 halophyte names, see archived data files). We chose this identification method because the published phylogeny includes all angiosperm sequences on GenBank and is not restricted to the taxonomic classification on The Plant List [45]. We did not collapse infraspecific taxa to the species level for this analysis since the published phylogeny includes infraspecific taxa. We also identified whether the tips associated with each family were monophyletic in the phylogeny. In order to restrict the analysis to families with sufficiently large phylogenies and more than a few halophytes, we considered only families with 25 or more taxa included in the Smith *et al*. [12] phylogeny, of which at least six were recognized halophytes.

Under these criteria, we selected 22 families, including families with more halophytes than expected by chance, families with fewer halophytes than expected, and also families that fell within the expected number of halophytes for the family size. We first extracted sixteen families that were monophyletic in the Smith *et al*. [12] phylogeny. For each monophyletic family we extracted the family subtree from the phylogeny, which included all tips in the Smith *et al*. [12] phylogeny belonging to that family according to GenBank taxonomy. Next we extracted subtrees for families that met our selection criteria but were not strictly monophyletic in the Smith *et al.* [12] tree. For these families, we extracted a monophyletic family subtree by removing a small number of taxa that were assigned to the target family in GenBank taxonomy but did not fall into that family clade in the published phylogeny. In some cases we also excluded a small number of taxa within the target family clade that were assigned to a different family (see Additional file 1 for details on the names of taxa excluded from each family subtree). We then removed tips from the family subtrees that were not identified with standardized genus and species epithets. We excluded any tips with labels that included the taxonomic epithets “af”, “aff”, “cf” or “sp”. We also removed tips that represented hybrid taxa by identifying tip labels that included one genus and two specific epithets, as well as the word “hybrid” or where the two species names were separated by “x”. All family subtrees were extracted and analyzed with equal branch lengths. Polytomies in the family subtrees were randomly resolved using the multi2di function in the R package ‘ape’ [52].

#### Metrics for analyzing phylogenetic patterns

For each family subtree we used two metrics to assess the phylogenetic pattern of halophytes within the family. Specifically, we aimed to test whether any of these families showed the same evolutionary pattern of salt tolerance as the grass family, having (1) many shallow inferred origins of salt tolerance, near the tips of the phylogeny, which gave rise to small clades of halophytes, and (2) origins that were spread across the phylogeny, occurring in many different lineages [8]. To detect these patterns in our sample of families, we used two metrics: the number of tips per origin (NoTO) and the sum of sister clade differences, a measure of phylogenetic clustering (SSCD) [53, 54].

The number of tips per origin (NoTO) metric is used to test whether, given the number of halophyte taxa in the tree (tips), there are significantly more inferred origins of salt tolerance, and thus smaller clades of halophytes, than we would expect under a Brownian motion model of trait evolution. It is possible that salt tolerance has been gained and lost multiple times and that salt tolerant lineages have since gone extinct [35]. Here we only infer gains of salt tolerance that lead to extant salt tolerant species, as our aim is to infer the minimum number of independent gains needed to explain the extant phylogenetic distribution of halophytes. Our aim was to compare the observed taxonomic and phylogenetic distribution of halophytes to a null model of trait evolution using trait reconstruction techniques. To estimate the NoTO for each family subtree, we inferred the minimum number of gains and losses of salt tolerance required to explain the observed topological distribution of halophyte tips, and then calculated the average number of halophyte tips per inferred gain. A shallow, scattered distribution of halophytes, where most halophyte species arise from gains near the tips of the phylogeny and most gains lead to only a few halophyte species, will have a low value for NoTO.

To generate a null distribution of the expected number of gains given a number of known halophytes, we used established methods to simulate salt tolerance as a continuous trait on each family subtree using a Brownian motion model [54, 55]. We then used an appropriate threshold to convert the continuous trait to a binary one, such that the number of halophyte tips in the simulated tree was equal to the number of identified halophytes in the family subtree. We repeated this process 1000 times to generate a null distribution of NoTO values, specific to the observed number of halophytes and the size of the subtree for comparison with the observed NoTO value for each family subtree. To generate a *p*-value for each family, we calculated the proportion of simulated trees that had a NoTO value lower than or equal to the observed. *P*-values less than or equal to 0.05 represent significantly smaller clades of halophytes than expected under Brownian motion.

The sum of sister clade differences (SSCD) metric describes the degree of phylogenetic clustering of halophytes. We used the method of calculating SSCD described by Fritz and Purvis [54]. Each tip was coded as 1 if it was on the halophyte list and 0 if it was not. Each internal node in the family subtree was assigned a trait state using the mean of the descendant node or tip states (e.g., if one descendant was state 1 and the other was 0, the node value was 0.5). The SSCD was calculated as the sum of the absolute difference between trait states of each pair of sister nodes or tips over the whole tree. If gains of salt tolerance were scattered across the phylogeny, each giving rise to one or few halophyte species in a small clade of salt tolerant taxa, we would expect a large SSCD value. We compared the observed SSCD of each family subtree to the SSCD for 1000 traits generated under Brownian motion on the subtree, using the same method for generating the null distribution for the NoTO value. The *p*-value was the proportion of simulated trees that have higher SSCD values than the observed. *P*-values less than or equal to 0.05 indicated that salt tolerance is significantly scattered on the phylogeny compared to a Brownian motion trait.

We conducted correlation tests to assess whether estimates of NoTO and SSCD were influenced by incomplete sampling in the family subtrees or by the proportion of halophytic taxa in the family subtrees. Since NoTO, SSCD, and sampling values represent proportions, we used a non-parametric correlation test, Kendall’s tau [56].

### Availability of supporting data

The data set supporting the results of this article is available in the Dryad repository [57]. http://dx.doi.org/10.5061/dryad.n64kk.

## Additional file

Additional file 1:
**Supplemental material.**
