# Supplementary material for: Salt tolerance is evolutionarily labile in a diverse set of angiosperm families
Source: BMC Evol Biol. 2015 May 19;15:90. doi: 10.1186/s12862-015-0379-0 (PMC4436861; doi:10.1186/s12862-015-0379-0)
Supplement: Additional file 1: — Supplemental material. [file 12862_2015_379_MOESM1_ESM.docx]

Supplemental material for “**Salt tolerance is evolutionarily labile in a diverse set of angiosperm families**”

C. Moray, X. Hua, L. Bromham

**1. Composition of halophyte list**

**2. Identification of angiosperm families for taxonomic analysis**

**3. Extraction of non-monophyletic family subtrees**

**Table S1**: Results of taxonomic analysis identifying families with more or fewer halophytes than expected for the 146 families with one or more halophytes

**Figure S1**: Family subtrees

**1. Composition of halophyte list**

We started with a list of halophytes from Menzel and Lieth (2003), then added halophytes included in lists of salt tolerant species from more recent publications. We then verified the list for synonymy, as described in the Materials and Methods section. Here we outline which species we included in our halophyte list from each source, since the definitions and terminology used to identify salt tolerant taxa differs between sources. Because information on specific levels of salinity tolerance is rare, in general we included species that were listed as salt tolerant based on observational evidence (that is, reported as being able to complete their life cycle under saline conditions). This means that we may have included species with relatively low levels of salt tolerance, or tolerant to external and occasional exposure to salinity (i.e., salt spray), so it is possible that not all species in our compiled halophyte list are able to grow in highly salt-affected soil.

We added taxa identified as halophytes from studies in Turkey (Guvensen *et al.*, 2006; Özturk *et al.*, 2008), China (Zhao *et al.*, 2010), Pakistan (Khan & Qaiser, 2006). From Dagar and Gurbachan

(2007), we added all species listed as true halophytes, facultative halophytes and glycophytes/transitional halophytes, since their definition of glycophytes/transitional halophytes includes species that are able to grow in saline soils.

**2. Identification of Angiosperm families for taxonomic analysis**

We identified 411 unique families based on the APG website (Stevens, 2001), whereas some sources have identified 413 (Haston *et al.*, 2009). For this analysis we started with the 413 families listed by the LAPG III (Haston *et al.*, 2009) and checked all family names against the APG III website (Stevens, 2001). During this search we found that Aristolochiaceae and Lactoridaceae are considered one family by the APG III website and that Buxaceae and Haptanthaceae are also considered synonyms (Stevens, 2001). Here we considered these families as synonyms, reducing the number of angiosperm families considered in this analysis from 413 to 411. We also recognized Ripogonaceae (Haston *et al.*, 2009) as an alternative spelling of Rhipogonaceae (APG III, 2009).

**3. Extraction of non-monophyletic family subtrees**

Based on the selection criteria chosen for the phylogenetic analysis (see Methods), we needed to extract some subtrees for families that were not monophyletic in the published angiosperm phylogeny (Smith *et al.*, 2011). In general we extracted all tips associated with each target family, excluding a small number of tips that were either nested within clades of other families or tips from other families that were nested within the clade of the target family. Here we list details on each non-monophyletic family subtree, referring to specific tip numbers associated with the original published phylogeny. For the Asteraceae subtree, we excluded one Asteraceae tip that was nested within the Campanulaceae clade (tip number 10079). For Brassicaceae we excluded five tips that were in the Capparaceae clade (42135, 42136, 42145, 42168, 42171). For Euphorbiaceae we excluded four Peraceae tips that were nested in the Euphorbiaceae clade (36371:36374). For Lamiaceae we excluded one tip that was in the Verbanaceae clade (21536), and one Orobanchaceae (24129) that was in the Lamiaceae clade. For Rosaceae we excluded one tip that was in Ranunculaceae (27875). For Rubiaceae we excluded one tip that was in the Clusiaceae clade (35882).

Table S1: Results of taxonomic analysis identifying families with more or fewer halophytes than expected for the 146 families with one or more halophytes. Family and order names come from the APG III website (Stevens, 2001). Genera and species represent the mean number of estimated genera and species in each family according to the APG III website (Stevens, 2001). Observed number and percentage of halophytes are based on the family affiliation of each accepted species in the halophyte list according to The Plant List (The Plant List, 2010). For Zosteraceae there were more observed halophytes (17) than estimated species in the family (14) since the mean species estimates come from the APG III website and the accepted species names in the halophyte list were confirmed with The Plant List (2010). For the analysis we considered Zosteraceae to have 100% halophytes, since the test is not valid when there are more halophytes than total species. *P*-values represent whether each family has more or fewer halophytes than expected under a binomial distribution (see Methods).

| **Order name**  **(APG III)** | **Family name**  **(APG III)** | **Species** | **Observed # halophytes** | **Observed % halophytes** | ***p*-value**  **fewer** | ***p*-value**  **more** | **Pattern** |
| --- | --- | --- | --- | --- | --- | --- | --- |
| Alismatales | Alismataceae | 88 | 3 | 3.4 | 0.99 | 0.06 |  |
| - | Aponogetonaceae | 43 | 1 | 2.3 | 0.93 | 0.36 |  |
| - | Araceae | 4759 | 4 | 0.1 | 0.00 | 1.00 | fewer |
| - | Butomaceae | 1 | 1 | 100.0 | 1.00 | 0.01 | more |
| - | Cymodoceaceae | 16 | 15 | 93.8 | 1.00 | 0.00 | more |
| - | Hydrocharitaceae | 116 | 22 | 19.0 | 1.00 | 0.00 | more |
| - | Juncaginaceae | 15 | 3 | 20.0 | 1.00 | 0.00 | more |
| - | Posidoniaceae | 9 | 3 | 33.3 | 1.00 | 0.00 | more |
| - | Potamogetonaceae | 102 | 6 | 5.9 | 1.00 | 0.00 | more |
| - | Ruppiaceae | 6 | 2 | 33.3 | 1.00 | 0.00 | more |
| - | Zosteraceae | 14 | 14 | 100.0 | 1.00 | 0.00 | more |
| Apiales | Apiaceae | 3780 | 33 | 0.9 | 0.19 | 0.85 |  |
| - | Araliaceae | 1450 | 3 | 0.2 | 0.00 | 1.00 | fewer |
| Arecales | Arecaceae | 2361 | 35 | 1.5 | 0.98 | 0.02 | more |
| Asparagales | Amaryllidaceae | 1605 | 15 | 0.9 | 0.41 | 0.68 |  |
| - | Asparagaceae | 2480 | 22 | 0.9 | 0.28 | 0.79 |  |
| - | Iridaceae | 2025 | 10 | 0.5 | 0.01 | 1.00 | fewer |
| - | Orchidaceae | 22075 | 3 | 0.0 | 0.00 | 1.00 | fewer |
| - | Xanthorrhoeaceae | 900 | 3 | 0.3 | 0.02 | 1.00 | fewer |
| Asterales | Asteraceae | 23600 | 267 | 1.1 | 0.94 | 0.07 |  |
| - | Calyceraceae | 60 | 1 | 1.7 | 0.87 | 0.46 |  |
| - | Goodeniaceae | 430 | 6 | 1.4 | 0.84 | 0.29 |  |
| Brassicales | Bataceae | 2 | 2 | 100.0 | 1.00 | 0.00 | more |
| - | Brassicaceae | 3710 | 38 | 1.0 | 0.53 | 0.54 |  |
| - | Capparaceae | 480 | 10 | 2.1 | 0.99 | 0.03 | more |
| - | Cleomaceae | 300 | 7 | 2.3 | 0.99 | 0.04 | more |
| - | Resedaceae | 75 | 5 | 6.7 | 1.00 | 0.00 | more |
| - | Salvadoraceae | 11 | 4 | 36.4 | 1.00 | 0.00 | more |
| Caryophyllales | Aizoaceae | 2035 | 45 | 2.2 | 1.00 | 0.00 | more |
| - | Amaranthaceae | 2275 | 507 | 22.3 | 1.00 | 0.00 | more |
| - | Anacampserotaceae | 32 | 1 | 3.1 | 0.96 | 0.28 |  |
| - | Basellaceae | 19 | 2 | 10.5 | 1.00 | 0.02 | more |
| - | Cactaceae | 1866 | 11 | 0.6 | 0.03 | 0.98 | fewer |
| - | Caryophyllaceae | 2200 | 25 | 1.1 | 0.73 | 0.34 |  |
| - | Didiereaceae | 16 | 2 | 12.5 | 1.00 | 0.01 | more |
| - | Frankeniaceae | 90 | 15 | 16.7 | 1.00 | 0.00 | more |
| - | Gisekiaceae | 5 | 1 | 20.0 | 1.00 | 0.05 |  |
| - | Halophytaceae | 1 | 1 | 100.0 | 1.00 | 0.01 | more |
| - | Lophiocarpaceae | 6 | 1 | 16.7 | 1.00 | 0.06 |  |
| - | Molluginaceae | 87 | 4 | 4.6 | 1.00 | 0.01 | more |
| - | Nyctaginaceae | 395 | 9 | 2.3 | 0.99 | 0.02 | more |
| - | Plumbaginaceae | 836 | 62 | 7.4 | 1.00 | 0.00 | more |
| - | Polygonaceae | 1110 | 40 | 3.6 | 1.00 | 0.00 | more |
| - | Portulacaceae | 70 | 11 | 15.7 | 1.00 | 0.00 | more |
| - | Sarcobataceae | 2 | 1 | 50.0 | 1.00 | 0.02 | more |
| - | Simmondsiaceae | 1 | 1 | 100.0 | 1.00 | 0.01 | more |
| - | Stegnospermataceae | 3 | 1 | 33.3 | 1.00 | 0.03 | more |
| - | Talinaceae | 27 | 2 | 7.4 | 1.00 | 0.03 | more |
| - | Tamaricaceae | 90 | 55 | 61.1 | 1.00 | 0.00 | more |
| Celastrales | Celastraceae | 1400 | 8 | 0.6 | 0.05 | 0.98 | fewer |
| Ceratophyllales | Ceratophyllaceae | 6 | 1 | 16.7 | 1.00 | 0.06 |  |
| Commelinales | Commelinaceae | 652 | 4 | 0.6 | 0.20 | 0.90 |  |
| - | Pontederiaceae | 33 | 3 | 9.1 | 1.00 | 0.00 | more |
| Cornales | Loasaceae | 265 | 1 | 0.4 | 0.24 | 0.94 |  |
| Cucurbitales | Cucurbitaceae | 960 | 14 | 1.5 | 0.92 | 0.13 |  |
| Dilleniales | Dilleniaceae | 355 | 1 | 0.3 | 0.12 | 0.97 |  |
| Dipsacales | Caprifoliaceae | 890 | 2 | 0.2 | 0.01 | 1.00 | fewer |
| Ericales | Ebenaceae | 548 | 4 | 0.7 | 0.33 | 0.82 |  |
| - | Ericaceae | 3995 | 1 | 0.0 | 0.00 | 1.00 | fewer |
| - | Lecythidaceae | 310 | 4 | 1.3 | 0.78 | 0.40 |  |
| - | Primulaceae | 2590 | 14 | 0.5 | 0.01 | 1.00 | fewer |
| - | Sapotaceae | 1100 | 2 | 0.2 | 0.00 | 1.00 | fewer |
| - | Tetrameristaceae | 5 | 1 | 20.0 | 1.00 | 0.05 |  |
| Fabales | Fabaceae | 19500 | 243 | 1.2 | 1.00 | 0.00 | more |
| - | Polygalaceae | 965 | 3 | 0.3 | 0.01 | 1.00 | fewer |
| - | Surianaceae | 8 | 1 | 12.5 | 1.00 | 0.08 |  |
| Fagales | Betulaceae | 145 | 1 | 0.7 | 0.56 | 0.78 |  |
| - | Casuarinaceae | 95 | 12 | 12.6 | 1.00 | 0.00 | more |
| Gentianales | Apocynaceae | 4555 | 43 | 0.9 | 0.31 | 0.74 |  |
| - | Gentianaceae | 1655 | 13 | 0.8 | 0.20 | 0.87 |  |
| - | Loganiaceae | 420 | 1 | 0.2 | 0.07 | 0.99 |  |
| - | Rubiaceae | 13150 | 13 | 0.1 | 0.00 | 1.00 | fewer |
| Geraniales | Geraniaceae | 805 | 1 | 0.1 | 0.00 | 1.00 | fewer |
| Lamiales | Acanthaceae | 4000 | 18 | 0.5 | 0.00 | 1.00 | fewer |
| - | Bignoniaceae | 800 | 9 | 1.1 | 0.69 | 0.44 |  |
| - | Lamiaceae | 7173 | 27 | 0.4 | 0.00 | 1.00 | fewer |
| - | Linderniaceae | 195 | 2 | 1.0 | 0.67 | 0.60 |  |
| - | Orobanchaceae | 2060 | 17 | 0.8 | 0.21 | 0.85 |  |
| - | Pedaliaceae | 70 | 1 | 1.4 | 0.84 | 0.52 |  |
| - | Phrymaceae | 188 | 4 | 2.1 | 0.95 | 0.13 |  |
| - | Plantaginaceae | 1900 | 34 | 1.8 | 1.00 | 0.00 | more |
| - | Scrophulariaceae | 1800 | 15 | 0.8 | 0.24 | 0.83 |  |
| - | Verbenaceae | 918 | 15 | 1.6 | 0.97 | 0.06 |  |
| Laurales | Hernandiaceae | 55 | 2 | 3.6 | 0.98 | 0.11 |  |
| - | Lauraceae | 2500 | 2 | 0.1 | 0.00 | 1.00 | fewer |
| Liliales | Colchicaceae | 245 | 2 | 0.8 | 0.54 | 0.72 |  |
| - | Liliaceae | 610 | 1 | 0.2 | 0.01 | 1.00 | fewer |
| Magnoliales | Annonaceae | 2220 | 1 | 0.0 | 0.00 | 1.00 | fewer |
| Malpighiales | Bonnetiaceae | 35 | 1 | 2.9 | 0.95 | 0.30 |  |
| - | Chrysobalanaceae | 460 | 1 | 0.2 | 0.05 | 0.99 | fewer |
| - | Clusiaceae | 595 | 2 | 0.3 | 0.06 | 0.98 |  |
| - | Elatinaceae | 35 | 7 | 20.0 | 1.00 | 0.00 | more |
| - | Euphorbiaceae | 5735 | 42 | 0.7 | 0.01 | 0.99 | fewer |
| - | Hypericaceae | 560 | 1 | 0.2 | 0.02 | 1.00 | fewer |
| - | Linaceae | 300 | 4 | 1.3 | 0.80 | 0.37 |  |
| - | Phyllanthaceae | 1745 | 9 | 0.5 | 0.02 | 0.99 | fewer |
| - | Putranjivaceae | 210 | 1 | 0.5 | 0.36 | 0.89 |  |
| - | Rhizophoraceae | 149 | 19 | 12.8 | 1.00 | 0.00 | more |
| - | Salicaceae | 1010 | 6 | 0.6 | 0.10 | 0.95 |  |
| Malvales | Malvaceae | 4225 | 56 | 1.3 | 0.97 | 0.04 | more |
| - | Neuradaceae | 10 | 1 | 10.0 | 1.00 | 0.10 |  |
| - | Thymelaeaceae | 891 | 3 | 0.3 | 0.02 | 0.99 | fewer |
| Myrtales | Combretaceae | 500 | 12 | 2.4 | 1.00 | 0.01 | more |
| - | Lythraceae | 620 | 21 | 3.4 | 1.00 | 0.00 | more |
| - | Melastomataceae | 5005 | 1 | 0.0 | 0.00 | 1.00 | fewer |
| - | Myrtaceae | 4620 | 47 | 1.0 | 0.50 | 0.56 |  |
| - | Onagraceae | 656 | 6 | 0.9 | 0.48 | 0.67 |  |
| Nymphaeales | Nymphaeaceae | 58 | 3 | 5.2 | 1.00 | 0.02 | more |
| Oxalidales | Oxalidaceae | 770 | 2 | 0.3 | 0.01 | 1.00 | fewer |
| Pandanales | Pandanaceae | 885 | 11 | 1.2 | 0.79 | 0.31 |  |
| Picramniales | Picramniaceae | 49 | 1 | 2.0 | 0.91 | 0.40 |  |
| Piperales | Piperaceae | 3615 | 1 | 0.0 | 0.00 | 1.00 | fewer |
| - | Saururaceae | 6 | 1 | 16.7 | 1.00 | 0.06 |  |
| Poales | Bromeliaceae | 1770 | 2 | 0.1 | 0.00 | 1.00 | fewer |
| - | Cyperaceae | 5430 | 121 | 2.2 | 1.00 | 0.00 | more |
| - | Flagellariaceae | 4 | 1 | 25.0 | 1.00 | 0.04 | more |
| - | Juncaceae | 430 | 22 | 5.1 | 1.00 | 0.00 | more |
| - | Poaceae | 11160 | 335 | 3.0 | 1.00 | 0.00 | more |
| - | Restionaceae | 500 | 2 | 0.4 | 0.11 | 0.97 |  |
| - | Typhaceae | 25 | 9 | 36.0 | 1.00 | 0.00 | more |
| Proteales | Nelumbonaceae | 2 | 1 | 50.0 | 1.00 | 0.02 | more |
| Ranunculales | Menispermaceae | 442 | 3 | 0.7 | 0.33 | 0.83 |  |
| - | Papaveraceae | 760 | 3 | 0.4 | 0.05 | 0.98 | fewer |
| - | Ranunculaceae | 2525 | 17 | 0.7 | 0.04 | 0.98 | fewer |
| Rosales | Elaeagnaceae | 45 | 3 | 6.7 | 1.00 | 0.01 | more |
| - | Moraceae | 1125 | 7 | 0.6 | 0.11 | 0.94 |  |
| - | Rhamnaceae | 925 | 6 | 0.6 | 0.16 | 0.91 |  |
| - | Rosaceae | 2520 | 9 | 0.4 | 0.00 | 1.00 | fewer |
| - | Ulmaceae | 35 | 1 | 2.9 | 0.95 | 0.30 |  |
| Santalales | Olacaceae | 57 | 1 | 1.8 | 0.88 | 0.45 |  |
| - | Santalaceae | 990 | 3 | 0.3 | 0.01 | 1.00 | fewer |
| Sapindales | Anacardiaceae | 873 | 7 | 0.8 | 0.32 | 0.79 |  |
| - | Meliaceae | 615 | 6 | 1.0 | 0.55 | 0.61 |  |
| - | Nitrariaceae | 16 | 8 | 50.0 | 1.00 | 0.00 | more |
| - | Rutaceae | 2070 | 5 | 0.2 | 0.00 | 1.00 | fewer |
| - | Sapindaceae | 1630 | 2 | 0.1 | 0.00 | 1.00 | fewer |
| - | Simaroubaceae | 110 | 1 | 0.9 | 0.69 | 0.68 |  |
| Saxifragales | Crassulaceae | 1370 | 2 | 0.1 | 0.00 | 1.00 | fewer |
| - | Cynomoriaceae | 2 | 1 | 50.0 | 1.00 | 0.02 | more |
| Solanales | Convolvulaceae | 1625 | 22 | 1.4 | 0.92 | 0.12 |  |
| - | Hydroleaceae | 12 | 1 | 8.3 | 0.99 | 0.12 |  |
| - | Solanaceae | 2460 | 41 | 1.7 | 1.00 | 0.00 | more |
| Unplaced Asterid I | Boraginaceae | 2755 | 37 | 1.3 | 0.95 | 0.07 |  |
| Vitales | Vitaceae | 850 | 4 | 0.5 | 0.06 | 0.98 |  |
| Zingiberales | Zingiberaceae | 1208 | 1 | 0.1 | 0.00 | 1.00 | fewer |
| Zygophyllales | Zygophyllaceae | 285 | 30 | 10.5 | 1.00 | 0.00 | more |

**Figure S1**: Family subtrees for the sample of 22 angiosperm families analysed. Origins of salt tolerance identified by maximum parsimony (see Methods) are marked on each family with black circles. Tips in the subtrees identified as halophytes are marked in black in the ring around the subtree.

Apiaceae

Arecaceae

Asteraceae

Goodeniaceae

Brassicaceae

Amaranthaceae

Tamaricaceae

Cucurbitaceae

Primulaceae

Casuarinaceae

Rubiaceae

Acanthaceae

Lamiaceae

Euphorbiaceae

Rhizophoraceae

Combretaceae


Lythraceae

Myrtaceae

Cyperaceae

Juncaceae

Poaceae

Rosaceae

**References**

Dagar, J.C. & Gurbachan, S. 2007. Biodiversity of saline and waterlogged environments: Documentation, utilization and management. *NBA Scientific Bulletin Number - 9*, National Biodiversity Authority, Chennai, Tamil Nadu, India, pp. 78.

Guvensen, A., Gork, G. & Özturk, M. 2006. An overview of the halophytes in Turkey. *Sabkha ecosystems*, pp. 9–30. Springer, Netherlands.

Haston, E., Richardson, J.E., Stevens, P.F., Chase, M.W. & Harris, D.J. 2009. The Linear Angiosperm Phylogeny Group (LAPG) III: A linear sequence of the families in APG III. *Bot. J. Linn. Soc.* **161**: 128–131.

Khan, M.A. & Qaiser, M. 2006. Halophytes of Pakistan: characteristics, distribution and potential economic usages. *Sabkha ecosystems*, pp. 129–153. Springer, Netherlands.

Menzel, U. & Lieth, H. 2003. *HALOPHYTE Database V. 2.0 update.* (H. Lieth & M. Mochtchenko, eds). Kluwer, Netherlands.

Özturk, M., Guvensen, A., Sakçali, S. & Gork, G. 2008. Halophyte plant diversity in the Irano-Turanian phytogeographical region of Turkey. In: *Biosaline Agriculture and High Salinity Tolerance*, pp. 141–155. Birkhäuser, Basel.

Smith, S.A., Beaulieu, J.M., Stamatakis, A. & Donoghue, M.J. 2011. Understanding angiosperm diversification using small and large phylogenetic trees. *Am. J. Bot.* **98**: 404–414.

Stevens, P.F. 2001. Angiosperm Phylogeny Website. Version 12, July 2012. http://www.mobot.org/MOBOT/research/ APweb/

The Plant List. 2010. The Plant List. http://theplantlist.org

Zhao, K., Song, J., Feng, G., Zhao, M. & Liu, J. 2010. Species, types, distribution, and economic potential of halophytes in China. *Plant Soil* **342**: 495–509.
